# Single-cell transcriptomic landscape of metabolic reprogramming in kidney allograft rejection

**DOI:** 10.3389/fimmu.2026.1755316

**Published:** 2026-08-03

**Authors:** Zhenan Zhang, Yi Zheng, Long Zheng, An He, Juntao Chen, Jinyu Luo, Ming Wang, Ming Cai

**Affiliations:** Department of Urology, the Second Affiliated Hospital, Zhejiang University, School of Medicine, Hangzhou, China

**Keywords:** chronic kidney transplant rejection, heterogeneity, kidney allograft rejection, metabolic profiles, single-cell RNA sequencing

## Abstract

**Introduction:**

Chronic rejection remains a major barrier to long-term survival after renal transplantation. However, our understanding of the transcriptional programs and metabolic alterations that shape the functions of human kidney cell populations during rejection is still limited.

**Objectives:**

To generate a comprehensive, cell type–specific transcriptomic map of metabolic states in healthy and transplanted human kidney samples.

**Methods:**

We performed single-cell RNA sequencing on 33 kidney samples, constructing an atlas comprising 82,661 cells.

**Results:**

Analysis of average metabolic pathway activity across cell subpopulations revealed distinct metabolic profiles. These differences provided insights into both unique abnormalities and shared features underlying various rejection states. We identified downregulation of mitochondrial programs in multiple non-proximal tubular cell populations compared with their counterparts in healthy kidneys. In contrast, both proximal tubular (PT) cells and T cells exhibited relatively elevated oxidative phosphorylation-related transcriptional scores, though the mechanisms driving this shift were specific to cell type and rejection status. Additionally, rejection-associated macrophages demonstrated altered amino acid metabolism, accompanied by enhanced IL-1 signaling.

**Conclusion:**

This single-cell atlas, together with a metabolically based stratification of chronic kidney transplant rejection, offers novel insights into the heterogeneous nature of rejection and provides valuable guidance for the development of targeted therapies.

## Graphical Abstract

**Figure:**
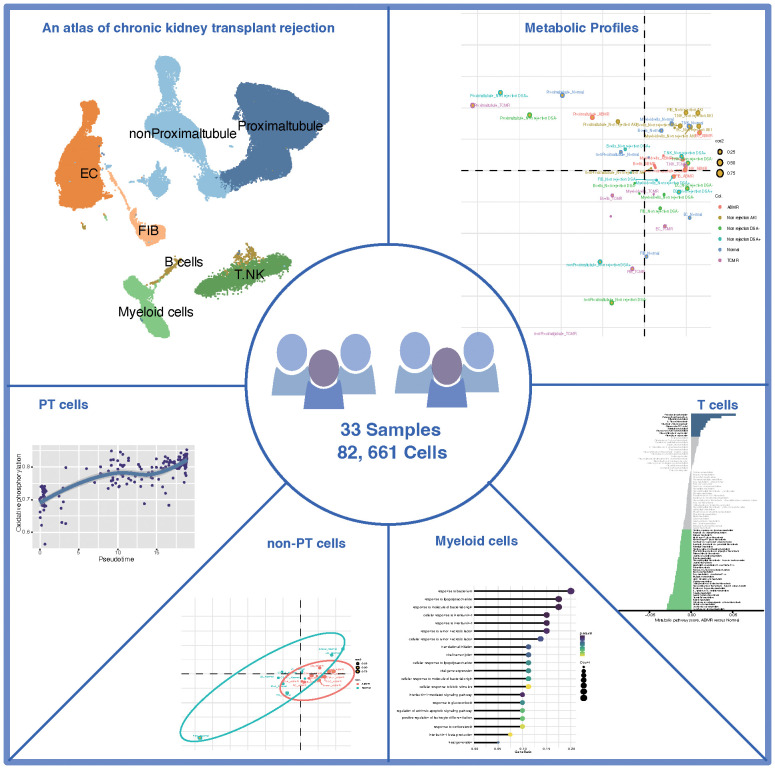

## Introduction

In clinical organ transplantation, kidney transplantation has consistently led in terms of the number of procedures performed. It is marked by an early start, rapid advancement, and strong efficacy, making it the most effective and preferred treatment option for patients with end-stage renal disease. Aside from transplants between identical twins, which bypass rejection, allogeneic kidney transplant recipients face potential rejection, including hyperacute, accelerated, acute, and chronic rejection. Chronic activated T cell-mediated rejection (TCMR) and chronic activated antibody-mediated rejection (ABMR) are categorized as the two main subtypes. With ongoing advancements in donor-recipient matching, hyperacute rejection has become increasingly rare. Similarly, with the development and combination of immunosuppressive therapies, acute rejection has declined. However, chronic rejection remains a key factor limiting the long-term survival of transplanted kidneys and is a primary cause of chronic kidney transplant failure.

Chronic rejection following kidney transplantation is a complex outcome driven by both immune and non-immune factors (1). Previous studies showed that *FCGR3A*+ monocytes expressed high levels of *CD47* and *LILR* genes, which enhanced paracrine signaling pathways and promoted T cell infiltration (2). Antigen-presenting cells recognized donor alloantigens and presented them on their surfaces, where they could be detected by T cells, contributing to allograft rejection, and resulting in interstitial fibrosis. Furthermore, B lymphocytes undergo antigen-driven proliferation, selection, and maturation, promoting B cell differentiation and antibody production (3). Immune cells might also interact with stromal cells, playing a pivotal role in driving pro-inflammatory and pro-fibrotic activities (4). However, there remained no consensus on effective treatments for chronic rejection, and several therapies targeting the humoral immune system, such as anti-CD20 monoclonal antibodies, have yet to show long-term efficacy (5). Further investigation into the intricate mechanisms underlying TCMR and ABMR, and the key regulatory molecules influencing its progression could support the development of new strategies to mitigate chronic kidney transplant rejection, providing substantial value for both scientific research and clinical practice.

The complex anatomy and function of the kidney made it challenging to understand the mechanisms driving the onset and progression of renal disease. However, genome-wide bulk mRNA profiling of kidney tissue offered a way to map the molecular basis of disease development and progression (6, 7). While single-cell RNA sequencing (scRNA-seq) initially faced obstacles in identifying molecular abnormalities and capturing cell type-specific transcriptional patterns, recent scRNA-seq studies have successfully characterized various kidney diseases. In healthy human kidneys, scRNA-seq revealed sex-based transcriptional programs and tissue-specific immune features (8). In lupus nephritis, an interferon response signature was detected in tubular cells, with expression levels correlating to disease severity and response to treatment (9). scRNA-seq of peripheral blood mononuclear cells (PBMCs) from patients with ABMR has mapped the transcriptomic landscape, highlighting strong interactions between T/B cells and neutrophils in ABMR pathogenesis (10), A single human kidney allograft biopsy with mixed rejection showed complex immune cell infiltrates and proinflammatory responses in parenchymal cells (11). Additionally, myofibroblasts (MyoF), a specialized subset of fibroblasts expressing extracellular matrix components like collagen, were found in higher numbers in the chronic kidney transplant rejection group, where elevated immune cell and MyoF levels correlated with increased rates of renal rejection and fibrosis.

Dynamic regulation of intracellular metabolism is a critical component of tissue homeostasis, and metabolic abnormalities can lead to pathological changes in the kidney. Studies have shown that the synthesis and breakdown of lipids are highly regulated in healthy renal tubular epithelial cells (12). Excessive accumulation of lipid droplets within these cells can trigger lipotoxicity, promoting fibrosis development (13). Furthermore, during acute kidney injury (AKI), studies show that T cells in the kidney have poor fatty acid oxidation and significant glycolytic activity; preventing glutamine metabolism in these cells can lessen the severity of AKI (14). These investigations revealed that metabolic anomalies could offer a fresh perspective on renal diseases. However, a comprehensive understanding of metabolic coadaptations across various cell types in chronic kidney transplant rejection remains lacking.

In this study, scRNA-seq approaches were employed to mapping cell metabolic atlases of healthy kidney, non-rejection AKI, non-rejection DSA-, non-rejection DSA+, ABMR, and TCMR. Through a comparative analysis of metabolic gene profiles, we aimed to uncover the unique characteristics of cell states, dynamics, and regulatory mechanisms by identifying the commonalities and differences between no-rejection and rejection conditions in kidney transplantation.

## Methods

### Single-cell RNA-Seq datasets acquired in this study

We obtained scRNA-seq data from 33 samples diagnosed with chronic kidney transplant rejection or from healthy controls, sourced from published studies. This dataset included single-cell transcriptome profiles for healthy controls (12 samples), non-rejection (NR) AKI group (2 samples), NR DSA negative group (NR, DSA-, 4 samples), NR positive group (NR, DSA+, 8 samples), ABMR (6 samples), and TCMR (1 sample). These data were retrieved from the European Bioinformatics Institute (https://www.ebi.ac.uk/) and the Gene Expression Omnibus (GEO; https://www.ncbi.nlm.nih.gov/geo) under accession numbers E-MTAB-12051 (2), GSE145927 (15), GSE131685 (2), and GSE202109 (16). Further details on the patient samples were provided in **Supplementary Table 1**.

### Data filtering

Quality control was conducted on cells through a filtering process based on three criteria: total unique molecular identifiers (UMIs) per cell, the number of detected genes per cell, and the percentage of mitochondrial gene counts per cell. Cells were selected if their UMI counts ranged from 200 to 50,000, the number of detected genes per cell fell within the 10–90% range of total detected levels, and mitochondrial gene counts were below 10%.

### Data integration

Following quality control, we normalized the raw counts with the NormalizeData function in Seurat (17), producing a log-normalized count matrix for downstream analyses. To proceed, 2,000 highly variable genes were identified using the FindVariableFeatures function with nfeatures = 2000. A principal component analysis (PCA) was then conducted to capture the main variation axes, resulting in a 50-component matrix generated by the RunPCA function. Using these 50 principal components and the top 2000 variable genes, we applied Harmony (18), an algorithm that aligns shared cell types across multiple datasets, to correct batch effects in scRNA-seq data. A convergence plot was produced with the RunHarmony function under default settings. Integration performance was quantitatively evaluated using the integration Local Inverse Simpson’s Index (iLISI). iLISI was calculated using the first 20 principal components before Harmony correction and the first 20 Harmony-corrected dimensions after integration, with dataset identity used as the batch label. Higher iLISI values indicate greater local mixing of cells from different datasets. The batch-corrected and integrated data from Harmony were subsequently used for additional scRNA-seq analyses.

### Dimension reduction and unsupervised clustering

We processed single-cell data for dimensionality reduction and unsupervised clustering following the Seurat workflow. For the remaining cells, unsupervised clustering was performed in Seurat with 20 harmonies in the runUMAP function and a resolution of 0.3 in the FindClusters function, resulting in the final 7 clusters used in this analysis. Marker transcripts for each cluster were identified with the FindAllMarkers function, with results detailed in **Supplementary Table 2**. A second round of dimensionality reduction and unsupervised clustering was performed to further characterize subsets of cell types following the same method. Cluster identities were manually assigned based on markers specific to each cell population.

### Sub-clustering

Each major cell population was subsetted from the integrated dataset and reprocessed independently for higher-resolution analysis. For each subset, data were normalized, highly variable genes were selected, and scaled expression matrices were subjected to principal component analysis. Batch effects were corrected using Harmony, with group1 as the integration variable. The first 15 Harmony dimensions were used for UMAP visualization and neighbor graph construction. Graph-based clustering was performed at a resolution of 0.2 for proximal tubular cells, endothelial cells, fibroblast/pericyte cells, myeloid cells, T/NK cells, and B cells, and at a resolution of 0.1 for non-proximal tubular cells.

### Calculation of metabolic pathway activity

A total of 2,913 metabolic genes were extracted from 85 KEGG pathways. The AUCell package (version 1.8.0) (19) utilizes a rank-based scoring approach to evaluate the activity levels of these pathways, generating a gene set activation score for each cell through the “Area Under the Curve” (AUC) function. AUCell scores represented the relative enrichment of pathway-associated transcripts within individual cells.

### Pathway analysis

Differentially expressed genes were detected using the FindAllMarkers function, applying cut-off thresholds of |FC| > 0.25 and an adjusted p-value < 0.05. Gene Ontology (GO) enrichment analysis for the differentially expressed genes (DEGs) was conducted using the clusterProfiler package (version 3.18.1) (20).

### Developmental trajectory inference

To analyze the development of PT6, we used the Monocle algorithm (version 2.6.4) (21), focusing on the top 378 signature genes identified by the differentialGeneTest function, applying filters of logFC > 1 and adjusted p-value < 0.05.

### Statistical analysis

Metabolic pathway activity was quantified at the single-cell level using AUCell based on curated KEGG metabolic gene sets. Comparisons of pathway activity scores between two groups were performed using the Wilcoxon rank-sum test, while comparisons among multiple groups were performed using the Kruskal–Wallis test, as appropriate. P values were adjusted for multiple testing using the Benjamini–Hochberg method, and adjusted P < 0.05 was considered statistically significant.

## Results

### Single-cell map of kidney allograft rejection

**Figure 1:**
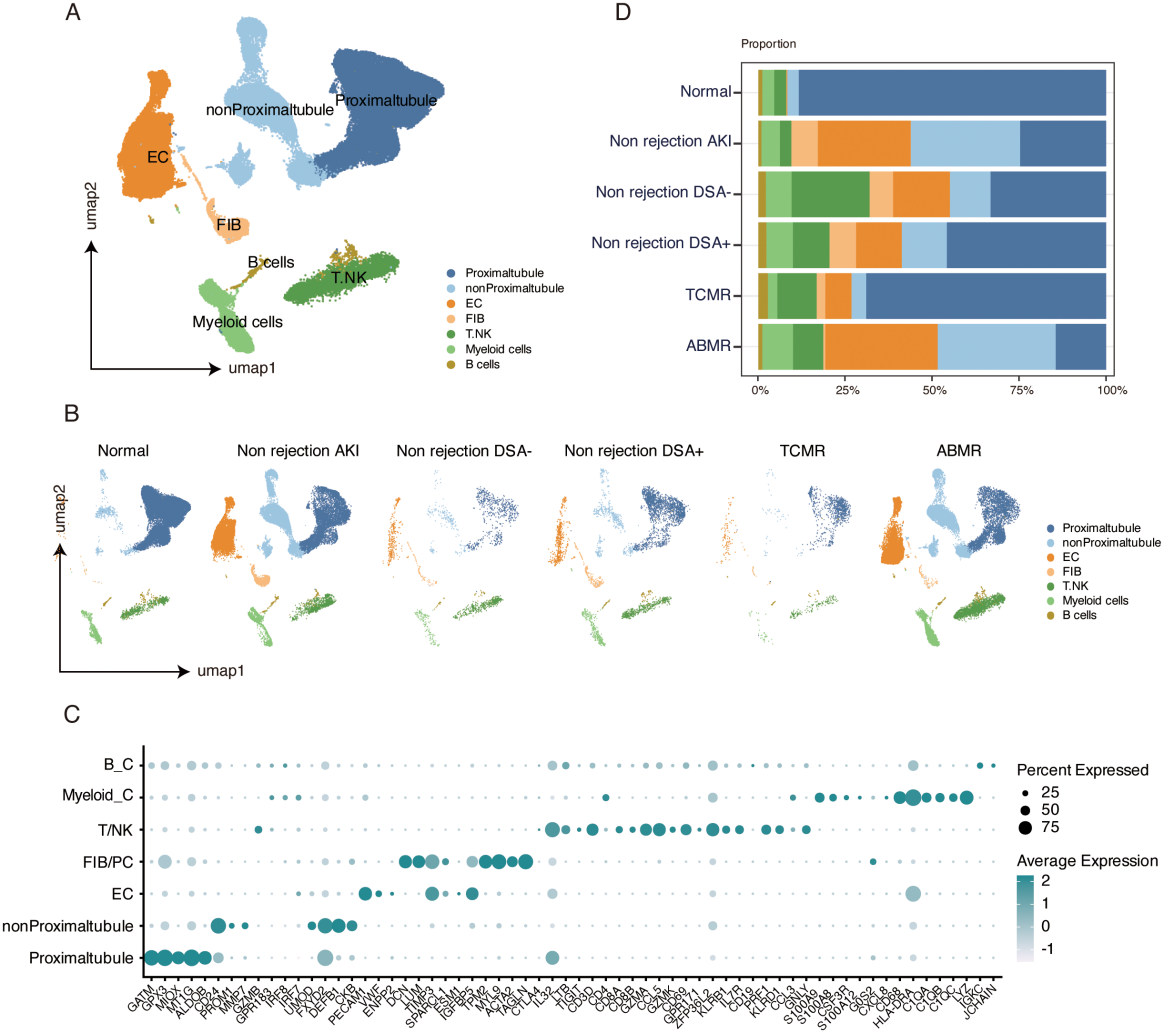
Classification of subtypes. **(A)** UMAP visualization of 7 cell clusters. **(B)** Uniform manifold approximation and projection (UMAP) representation of cell distribution for healthy controls, non-rejection AKI, non-rejection DSA-, non-rejection DSA+, ABMR, and TCMR. **(C)** Distribution of cell populations for healthy controls, non-rejection AKI, non-rejection DSA-, non-rejection DSA+, ABMR, and TCMR. **(D)** Dot plot displaying signature marker among cell types.

A total of 33 kidney samples were collected from healthy control subjects and patients with various conditions, including NR AKI, NR DSA-, NR DSA+, ABMR, and TCMR. Following dataset integration using a standardized workflow, we identified 82661 cells and initially classified them into 7 distinct cell clusters (**Figures 1A, B**).

Both visual and quantitative assessments revealed diverse cell-type abundances across samples (**Figure 1D**). We note that cell proportions inferred from scRNA-seq data are descriptive and may reflect not only biological variation but also differences in tissue sampling, dissociation efficiency, and cell capture. Within the profiled cells, PT cells were relatively enriched in TCMR samples but reduced in ABMR samples, whereas non-PT parenchymal populations showed a higher relative representation in ABMR. Cell-type composition derived from scRNA-seq was used as a descriptive measure and was not interpreted as a direct estimate of *in vivo* cell abundance.

As shown in **Figure 1C**, cell types were classified according to the characteristic expression of specific marker genes. Proximal tubular (PT) cells were identified by the expression of *GATM, GPX3, MIOX, MT1G, and ALDOB*. Non-proximal tubular cells were marked by the expression of *CD24, PROM1, and MMP7*. Fibroblast/pericyte cells (FIB/PC cells) showed strong expression of *DCN, LUM, ACTA2, and TAGLN*. Endothelial cells (EC) were identified by VWF and PECAM1 expression. Myeloid cells (Myeloid_C) displayed high levels of *CD68* and HLA, while T cells and natural killer (NK) cells were characterized by coordinated elevation of *CD3D, CD3G, CD3E, and GZMA*. B cells exhibited elevated expression of genes associated with immunoglobulin synthesis.

### Cell type-specific metabolic reprogramming

**Figure 2:**
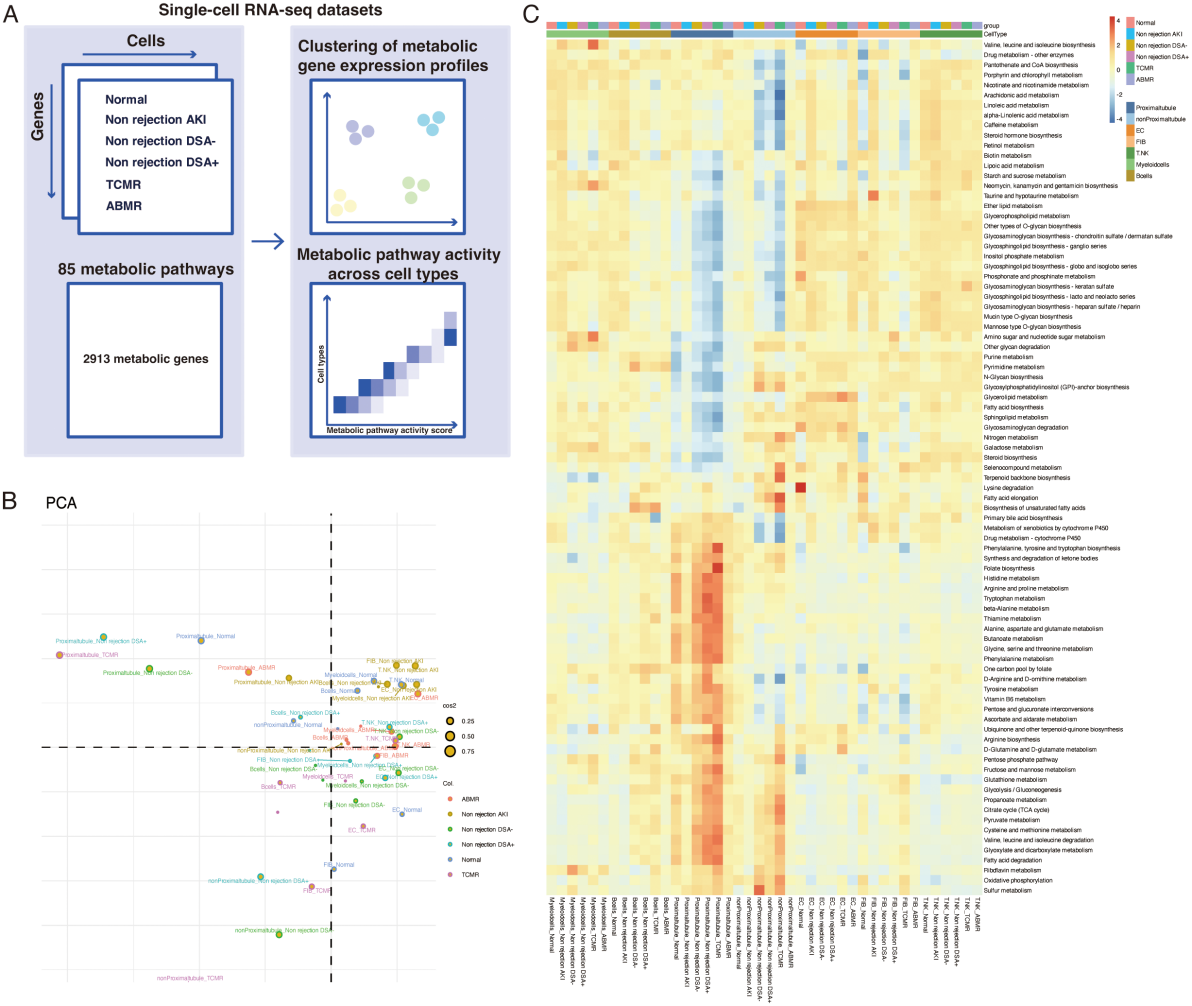
Workflow and metabolic heterogeneity. **(A)** Schematic representation of the scRNA-seq data analysis pipeline, combined with 85 metabolic pathways and 2913 metabolic genes. **(B)** PCA representing metabolic heterogeneities. **(C)** Heatmaps showing different expression patterns of metabolic genes among normal and transplanted kidneys.

Our next objective was to identify the key features of metabolic pathway variation across different cell types, with a specific focus on differences between TCMR and ABMR cells. To quantify metabolic pathway score, we used the AUCell method to calculate a pathway activity score. This method identifies cells with active gene signatures enriched among the expressed genes for each cell (**Figure 2A**).

To further identify whether these cellular metabolic changes are ABMR-specific or are common to kidney allograft rejection, we performed a Principal Component Analysis (PCA) based on the metabolic genes in KEGG and found that ABMR and TCMR had distinctive metabolic gene expression compared to normal samples, with the exception of EC cells. Non-rejection samples with DSA+, DSA-, and normal samples seemed to share relatively higher similarity (**Figure 2B**).

The clustered heatmap of average metabolic pathway activity for each cell subpopulation showed that each cell type had a distinct metabolic profile. Metabolic differences were more pronounced among ABMR, TCMR, and NR DSA+ samples than between different cell types, particularly in PT cells, non-PT cells, myeloid cells, and T cells (**Figure 2C**).

### Metabolism of PT cells is involved in ABMR

**Figure 3:**
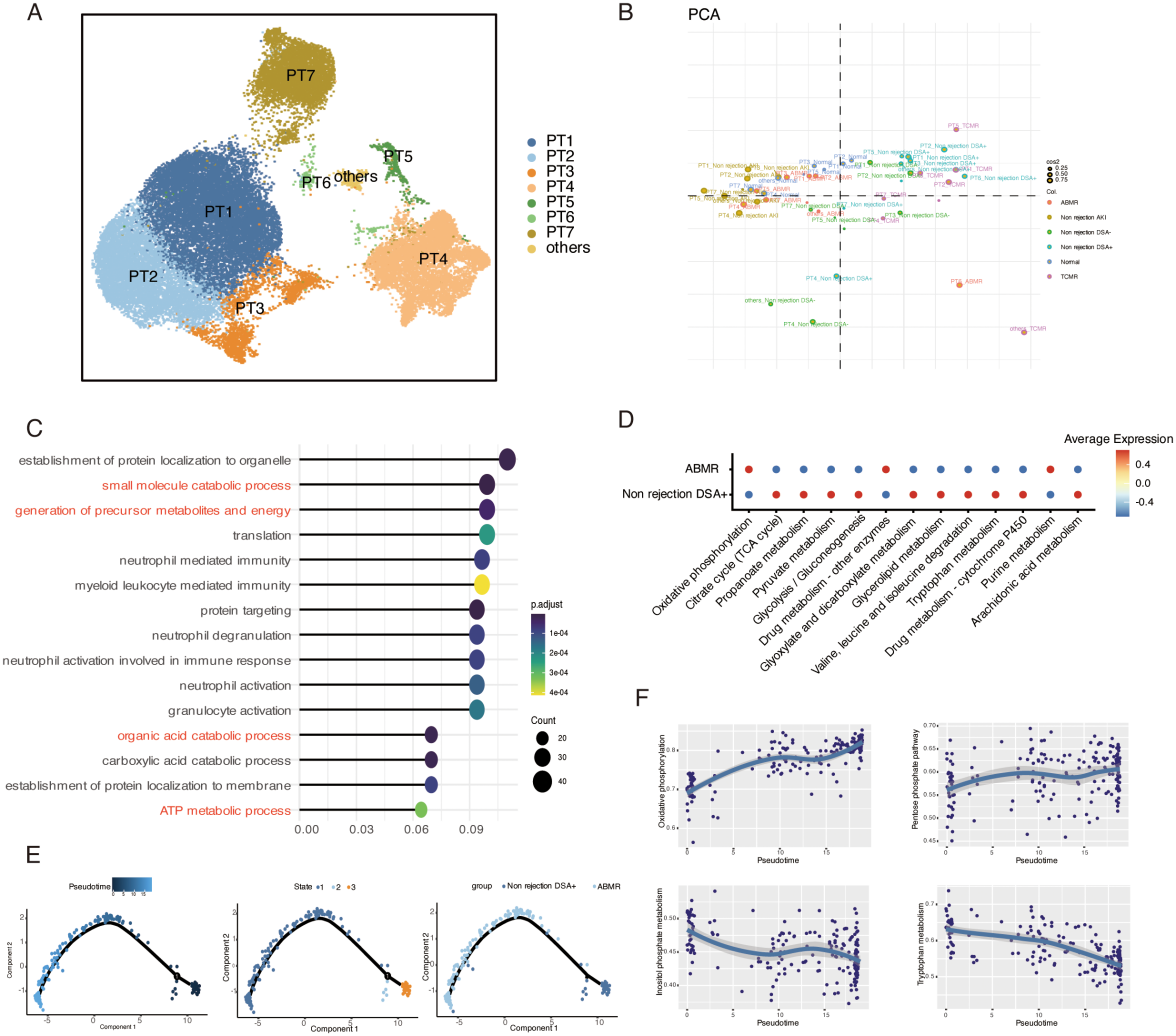
Metabolism of PT cells is involved in ABMR. **(A)** Cell-type-specific reference UMAPs for PT cells. **(B)** PCA representing metabolic heterogeneities among PT clusters. **(C)** GO enrichment analysis of differential gene expressions. **(D)** Metabolic pathways enriched in genes with highest contribution to the metabolic heterogeneities in PT6 cells between no-rejection (DSA+) and ABMR samples. **(E)** Distribution of PT6 on the pseudo-time trajectory. Cells were colored based on pseudotime, tissue types, and states. **(F)** Metabolic pathway activities along with the pseudotime.

A total of 7 PT clusters were identified (**Figure 3A**; **S1**), showing segment-specific separation. PT1, PT3, and PT6 were enriched for PT segment 1 (S1) marker *SLC5A2* and S1/S2-abundant genes (S*LC7A7, ANK2, SLC4A4, SLC6A19, SLC22A8*), while PT2 showed increased expression of S3-abundant genes (*DCXR, AGXT*). PT3 also exhibited high expression of dissociation stress-associated genes, along with general PT markers (*LRP2, CUBN, HSPA1A, HSP90B1*) and segment-specific PT genes. PT6 displayed elevated levels of *AL138963.4* and *SNHG29*, linked to cellular growth and proliferation. PT4,5, and 7 (*VIM* ^+^*S100A6*^+^) were similar to a putative regenerative PT population – called ‘scattered tubular cells’ (STC). PT4 was defined by their high expression levels of *VCAM1 and DCDC2*, PT5 was distinguished by the presence of *IGFBP5, RNASE1*, and *TIMP3*, and the expression of antioxidant markers such as *GSTP1* and *FABP3* characterized PT7.

Metabolic changes in PT6 were specific to ABMR, those in PT5 were specific to TCMR, and those in PT4 were specific to cases with no rejection (DSA+ or DSA-) (**Figure 3B**). We further analyzed the metabolic differences in PT6 cells between ABMR and other samples. First, differential gene expression (DEG) analysis revealed 477 differential genes (**Supplementary Table 3**), enriched in pathways including small molecule catabolic process, generation of precursor metabolites and energy, organic acid catabolic process, and ATP metabolic process (**Figure 3C**). Next, energy metabolism-related pathways differed significantly in ABMR and no rejection (DSA+) samples (**Figure 3D**). To further investigate the temporal coordination of metabolic remodeling with cell phenotype, we inferred a pseudotime axis representing PT6 progression in rejection and metabolic remodeling (**Figure 3E**). Pseudotime trajectory analysis revealed that cells from state 3 were primarily at the beginning of the projected timeline, while cells from states 1 and 2 appeared in the middle and end, respectively. Notably, subjects with no rejection (DSA+) were positioned in state 3 (**Figure 3F**), indicating that pseudotime correlated well with activation timing and was consistent across different trajectory algorithms and independent donors. Specifically, PT6 cells increased fibrosis score, as well as correlated with several energy metabolism-related pathways (**Supplementary Figure 2**). Notably, the increased fibrosis score observed in PT6 cells reflected activation of a fibrosis-associated transcriptional program within PT cells and should not be interpreted as equivalent to an increased proportion of fibroblasts at the tissue level. Altogether, our findings contributed to the increasing amount of evidence suggesting metabolic signaling as a key modulator of the development and maintenance of fibrosis; nevertheless, further functional research is required to substantiate this theory.

### Distinct activation of oxidative phosphorylation in ABMR non-PT cells

**Figure 4:**
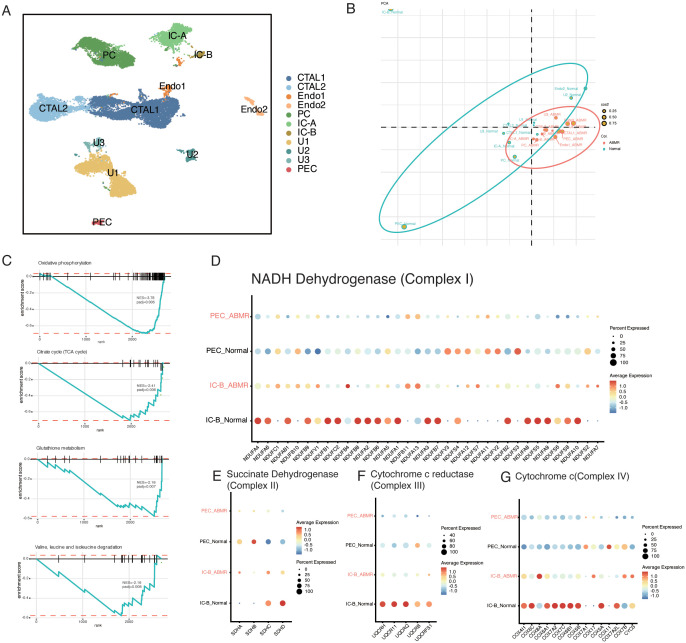
Distinct activation of oxidative phosphorylation in ABMR non-PT cells. **(A)** Cell-type-specific reference UMAPs for non-PT cells. **(B)** PCA illustrating metabolic heterogeneities between ABMR and normal samples. **(C)** GSEA of significantly up- or downregulated metabolic pathways in ABMR cells with corresponding NES and adjusted P values. **(D–G)** Dot plot visualization based on the average expression and percentage of cells expressing genes driving complexes I, II, III, and IV, respectively, in IC-B cells and PEC cells in healthy human (black) and ABMR samples (red).

A total of 11 non-PT parenchymal cell populations was identified (**Figure 4A**), including rare populations such as parietal epithelial cells. Cortical thick ascending limb (CTAL) was defined by their high expression levels of *UMOD*. Two CTAL subpopulations were identified based on the expression of *CLDN10* and *CLDN16*, representing cells with distinct paracellular cation-resorption preferences: *CLDN10*-dominant cells favor Na+ resorption, while *CLDN16*-dominant cells favor Ca2+ and Mg2+ resorption through their respective tight junctions. Among endothelial subpopulations (Endo1-2), we identified two populations (Endo1, Endo2) of peritubular capillary cells (*PLVAP*^+^*TMEM88*^+^*DNASE1L3*^+^), and expressed motility and angiogenesis markers *MARCKS, ACKR1, and SEMA3D*. Principal cell (PC) was identified by membrane-bound water channel genes (*AQP2*, *AQP3*) and *FXYD4* modulating the properties of the Na,K-ATPase. Intercalated cells (IC-A, IC-B) were important in regulating acid-base homeostasis (*SLC26A7, SLC4A1, SLC4A9, SLC26A4*). Parietal epithelial cells (PEC), which represented a reservoir of renal progenitors, were identified by *VIM, VCAM1*, and connective tissue growth factor (*CTGF*) expression (**Supplementary Figures 3A, B**).

PCA based on the metabolic genes in KEGG indicated that normal and ABMR samples had distinctive metabolic gene expression (**Figure 4B**). Given that PEC and IC-B showed the greatest metabolic variation across conditions, we started our investigation into metabolic rewiring with ABMR PEC and IC-B cells relative to normal cells. To determine if transcriptional variations within certain metabolic pathways may reveal the dysregulation of ABMR, we then calculated the enrichment scores of 85 metabolic pathways in each sample. When comparing ABMR samples to normal samples, a total of 18 metabolic pathways showed substantial dysregulation (2 pathways upregulated and 16 pathways downregulated) (|t value| > 1.5) (**Supplementary Figure 4A**). ABMR cells exhibited lower OXPHOS, citrate (TCA cycle), glutathione metabolism, and vitamin A and biosynthetic machinery (valine, leucine, and isoleucine degradation; glycine, serine, threonine metabolism; cysteine and methionine metabolism; beta-Alanine metabolism) (**Figure 4C**).

When compared to their counterparts in normal controls, components of the expression of complex I-encoding genes were downregulated to differing degrees in both the IC-B and PEC compartments (**Figure 4D**). The expression of various genes encoding complex I-driving proteins, such as *NDUFC1, NDUFB4, NDUFB10, NDUFB11, NDUFA12, NDUFA13, NDUFS7, NDUFS2, and NDUFA7*, showed the most pronounced reduction in IC-B cells in ABMR (**Figure 4D**). The expression of succinate dehydrogenase isoforms *SDHC* and *SDHD* was reduced by IC-B in ABMR while *SDHA* and *SDHB* were lowered by PEC in complex II, the second entrance into the ETC (**Figure 4E**). Complexes III and IV also showed variations in expression according to cell type in the ABMR when compared to healthy kidneys. Notably, the genes encoding complexes III and IV showed the most decline in expression in IC-B (**Figures 4F, G**). Thus, ETC-related gene expression showed a broadly reduced but compartment-specific pattern in ABMR. Rejection-associated cells did not dramatically change glycolysis in comparison to normal kidneys, even though we evaluated read coverage of glycolytic genes across IC-B and PEC compartments and found appropriate coverage (**Supplementary Figure 4B**). Taken together, this might indicate that IC-B and PEC linked to ABMR do not switch from glycolytic reliance to TCA and OXPHOS.

To better visualize the changes between IC-B and PEC cells in ABMR compared with normal tissue, we assessed the following pathways related to glutathione metabolism, and vitamin A and biosynthetic machinery, based on cells expressing genes related to each significantly altered pathway, alongside average expression (**Supplementary Figures 4C, D**). Compared to normal cells, cells from ABMR showed the greatest downregulation of amino acid degradation pathways. They included the metabolism of valine/leucine/isoleucine and cysteine/glutathione. The reduced expression of glutathione metabolism-related genes, including *GCLC* and *GCLM*, suggested a decline in glutathione synthesis capacity. This pattern may indicate reduced antioxidant potential in ABMR-associated stromal cells. In fact, we found that ABMR cells might have less antioxidant ability based on decreased glutathione metabolism.

### Lineage-specific roles in inflammatory and metabolic pathways in TCMR myeloid cells

**Figure 5:**
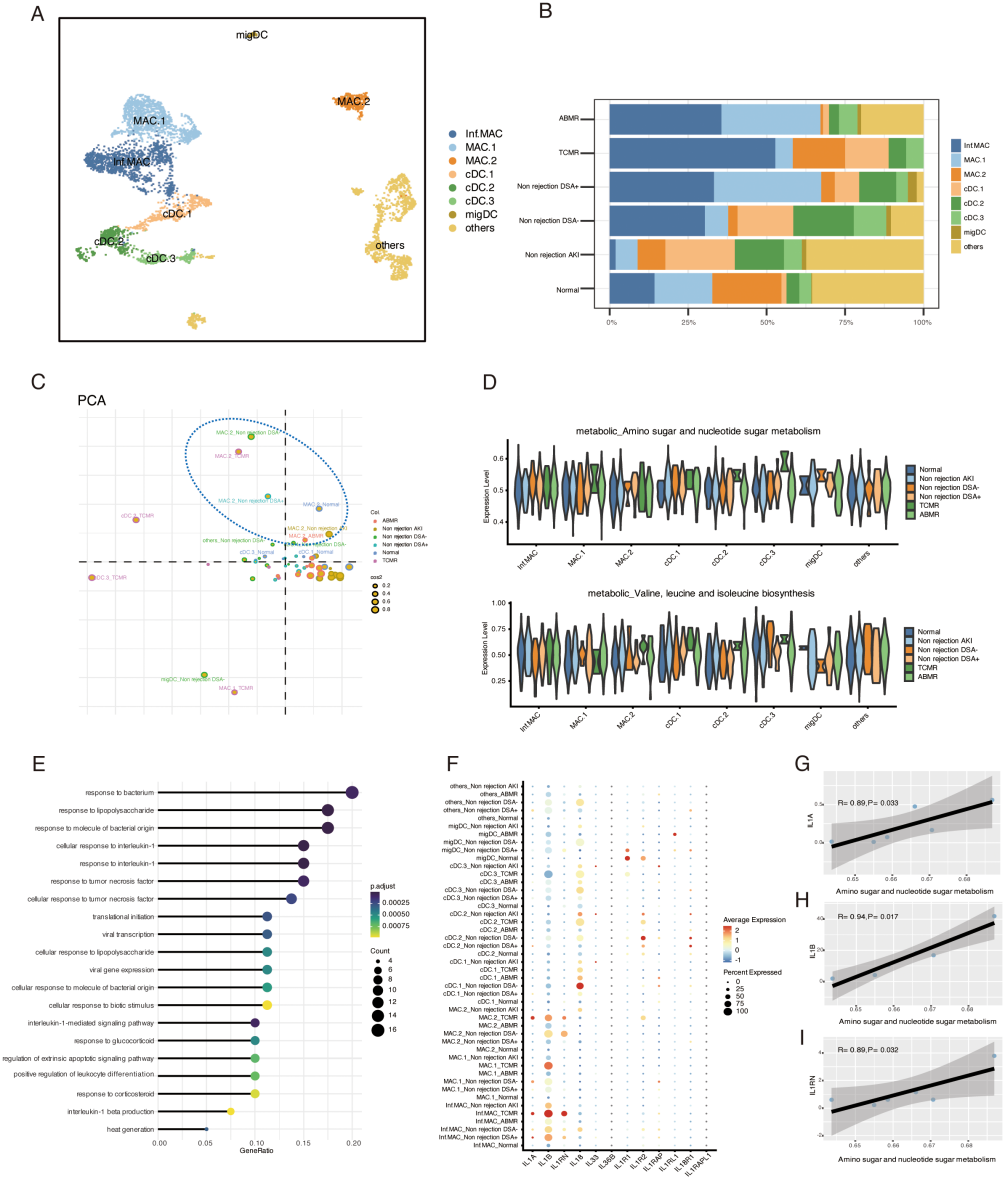
Lineage-specific roles in inflammatory and metabolic pathways in TCMR myeloid cells. **(A)** Cell-type-specific reference UMAPs for myeloid cells. **(B)** Proportion of cell clusters for each disease. **(C)** PCA showcasing metabolic diversity across myeloid cell types. **(D)** Violin plots of the pathway score of selected metabolism pathways comparing myeloid cells from transplanted kidney samples with those from healthy samples. **(E)** GO enrichment analysis of differential gene expressions. **(F)** Dot plot showing gene expressions of IL-1 signaling. **(G–I)** The correlation between *IL1A*, *IL1B*, and *IL1RN* expression and amino sugar nucleotide pathway activities.

We subclustered MAC_DC populations for higher resolution analysis and identified seven major lineages using canonical cell markers (**Figures 5A**; **S5**). Macrophage subsets (MAC.1, MAC.2) were distinguished by high expression of *CD68, CEBPB*, and *FCER1G*, while a third cluster (Inf.MAC) expressed *NLRP3* and showed an inflammatory, cytokine-enriched, and pro-angiogenic profile with markers like *IL1B* and *CCL3*. Dendritic cell (DC) populations were defined by MHCII molecules (*HLA-DRA, HLA-DRB1*) and included three classical subsets (cDC1, cDC2, cDC3) along with a mature subset (migDC). The abundances of different cell types varied among samples were observed. Inf.MAC cells showed notable expansion in TCMR samples but were reduced in ABMR samples. In contrast, MAC.1 cells in ABMR samples were increased, which constituted the highest proportion in non-rejection samples (DSA+) (**Figure 5B**).

Macrophages played important roles in healthy tissues, and many metabolic crosstalk features have been documented. To begin our analysis of myeloid cells, we first investigated significantly increased or decreased metabolic pathways in myeloid cells in rejection samples compared with in non-rejection samples (**Figure 5C**). In TCMR, we observed that amino sugar and nucleotide metabolism and valine, leucine, and isoleucine degradation activity were increased at the overall level. This is consistent with the next comparison among subpopulations pointing to altered pathway activity in MAC.2 (**Figure 5D**). Intrigued not only by the significant variation in MAC.2 proportions among samples, but we also found that MAC.2 exhibited a distinct metabolic pattern in sample conditions, as revealed by PCA.

As shown in **Figure 5E**, DEG analysis revealed distinct gene expression profiles in MAC.2, with gene ontology pathway enrichment analysis indicating roles in bacterial response, response to lipopolysaccharides, response to molecules of bacterial origin, and cellular response to interleukin 1. The IL-1 family, a group of cytokines crucial for immune regulation, includes key members like IL-1α, IL-1β, IL-18, and IL-33, which mediate inflammatory responses, immune regulation, and tissue repair. Elevated levels of *IL1A*, *IL1B*, and *IL1RN* were observed in MAC.2 and Inf.MAC cells in TCMR, while significant *IL1RL1* elevation was noted exclusively in migDCs in ABMR, suggesting a mixed inflammatory pattern of IL-1 cytokines in rejection samples (**Figure 5F**). Furthermore, metabolic pathways associated with IL1A, IL1B, and IL1RN were predominantly enriched in TCMR cells, suggesting that the intensity of inflammation in TCMR is linked to amino sugar and nucleotide metabolism (**Figures 5G–I**).

### Metabolic and functional heterogeneity of T Cells in rejection samples

**Figure 6:**
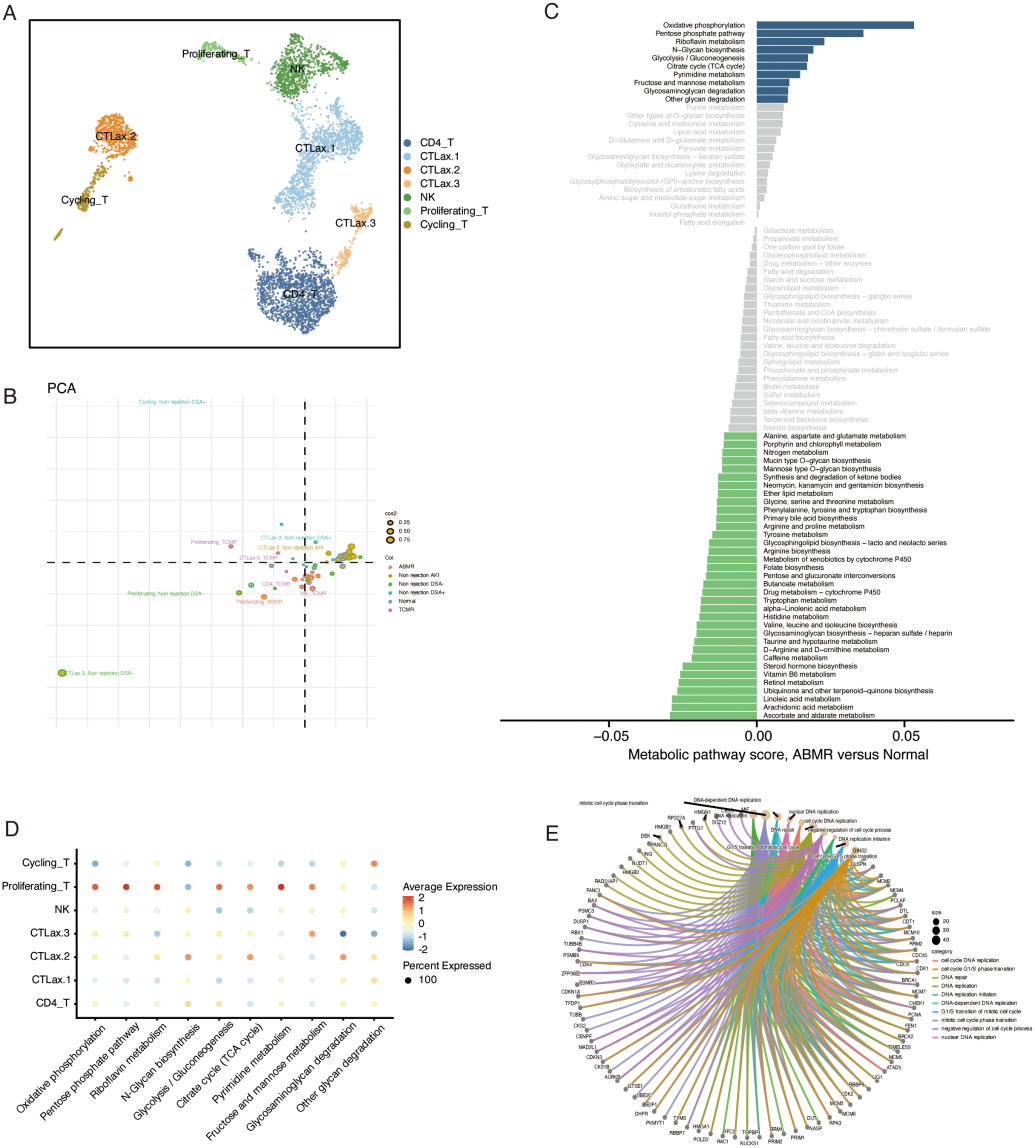
Metabolic and functional heterogeneity of T Cells in rejection samples. **(A)** Cell-type-specific reference UMAPs for T cells. **(B)** PCA showcasing metabolic diversity across T cell clusters. **(C)** Metabolic pathways enriched in ABMR or normal T cells. Significantly enriched pathways with GSEA p-value < 0.05 are highlighted in blue (higher in ABMR) or green (higher in normal samples). **(D)** Dot plot showing the top 10 metabolic pathways. **(E)** GO enrichment analysis of differential gene expressions.

As shown in **Figure 6A**, T cell clusters were assigned specific identities based on marker gene expression (**Supplementary Figures 6A, B**). In the CD8+ compartment, a major subset comprised activated cytotoxic T cells (CTLax1, CTLax2, CTLax3), characterized by high expression of *CCL5*, *NKG7*, and *GZMB*. Natural killer (NK) cells showed both cytotoxic markers (*NKG7*, *GZMA*, and *GZMB*) and NK-specific surface markers (*KLRB1*, *KLRD1*, *FCER1G*, *GNLY*), reflecting their cytotoxic role. CD4 and CD28 expression identified one CD4+ subset (CD4_T). The presence of *MKI67*, *UBE2C*, and *TOP2A* marked proliferating T cells. A small T cell cluster, termed Cycling_T, displayed elevated levels of cell cycle-related genes, including *CDKN1A* and *ZNF331*.

Intrigued by the marked variance in PCA analysis, we next assessed more globally the metabolic differences between rejection and normal samples by performing GSEA. ABMR samples showed significant upregulation of OXPHOS, pentose phosphate pathway, riboflavin metabolism, N-Glycan biosynthesis, glycolysis/gluconeogenesis, citrate cycle (TCA cycle), pyrimidine metabolism, fructose and mannose metabolism, glycosaminoglycan degradation, and other glycan degradation. In agreement with previous result of proliferating_T stood out with ABMR, whose pathways we observed to be highly regulated (**Figures 6B–D**). To interrogate master genes of proliferating T cells, go analysis was performed. The activity of t genes corresponds to regulon activity and pathway, where a higher score indicates a higher inferred level of genes, including *HMGA1* and *HMGB2* (**Figure 6E**). The strongest pathway for this analysis was cell cycle DNA replication.

Based on the observed dichotomous distribution pattern in PCA, we hypothesized that this activation of metabolism is the likely result of the TCMR and other no-rejection samples. The increase in bioenergetic pathways was accompanied by an activation in riboflavin metabolism. Further, analysis of the expression of bioenergetic pathways demonstrated pathways mainly activated in CTLax.3 and proliferating T cells (**Supplementary Figure 6C**). OXPHOS, Starch and sucrose metabolism, Pyrimidine metabolism, Cysteine and methionine metabolism, Lipoic acid metabolism, and Glyoxylate and dicarboxylate metabolism were increased in proliferating T cells, and neomycin, kanamycin and gentamicin biosynthesis, Glycolysis/Gluconeogenesis, fructose and mannose metabolism, Citrate cycle (TCA cycle), and Amino sugar and nucleotide sugar metabolism were activated in CTLax.3 (**Supplementary Figure 6D**). To further evaluate T-cell metabolic transcriptional programs in an independent cohort, we analyzed GSE189536, a kidney allograft biopsy single-cell/CITE-seq dataset containing transcriptomic libraries from 5 TCMR and 3 non-rejection biopsies. In all T cells, TCMR samples showed a directionally increased OXPHOS -related score compared with non-rejection samples (**Supplementary Figure 7**). These findings provided external support for TCMR-associated T-cell metabolic transcriptional remodeling, although the limited number of validation biopsies precluded definitive statistical validation. These results might provide a more detailed understanding of how the T cell metabolism and thus function and insight into how T cells works in TCMR.

## Discussion

With 82,661 single cells, we compared various cell types, proportions, and states to map the heterogeneity of transplanted and normal renal tissues, creating a comprehensive metabolic reference for kidney tissue. Previous research has used various approaches to investigate the mechanisms of different kidney transplant rejection responses. Given that immune-stromal interactions within the microenvironment contribute to transplant rejection, metabolic signaling can directly and indirectly influence immune cells. We aimed to gain new insights into metabolic abnormalities and shared characteristics between non-rejection and rejection conditions in kidney transplantation by analyzing single-cell metabolic gene expression profiles. We mapped gene expression changes associated with rejection and investigated metabolic alterations across all cellular compartments, thereby constructing a comprehensive metabolic atlas from healthy and kidney transplant tissues.

Spatially resolved metabolomics has uncovered region-specific metabolic changes in diabetic kidneys, such as altered glycolysis, dysregulated lipid metabolism, and imbalances in redox and osmotic homeostasis, each occurring in distinct regions (22). Although metabolites can be measured directly via liquid chromatography-mass spectrometry, this technique often requires strict sample storage conditions and is vulnerable to target degradation. As a result, alternative methods have been developed to evaluate metabolic status through gene expression analysis. Advances in single-cell sequencing now enable the detection of metabolic reprogramming at the single-cell level using bioinformatics. Metabolism directly informs the functional phenotypes of every cell present in the microenvironment (23). In our study, each cell type displayed a unique metabolic profile under various transplantation conditions.

In this study, we characterized the metabolic reprogramming of PT, non-PT, and immune cells in ABMR and TCMR samples. Our findings provided a resource atlas to understand the pathways utilized by stromal cells to maintain homeostasis and illustrated immune cell reprogramming and crosstalk in response to metabolic signals. Many metabolic studies have relied on *in vitro* systems, allowing for manipulation of media and metabolite levels and inclusion of select stromal and immune cells (24, 25). However, these conditions do not fully capture the physiological context in which the microenvironment drives metabolic dysregulation and competition for bioenergetic resources, nor do they address the complexities introduced by immunosuppression.

Accordingly, this resource atlas could also be of value to compare metabolic rewiring of the microenvironment in other non-rejection states, such as AKI, DSA-, and DSA+, but not only for researchers interested in the ABMR and TCMR. We suggested that energy metabolism is upregulated across multiple immune compartments in both ABMR and TCMR samples: proliferating T cells, CTLax.3. This marked increase in dependency on oxidative phosphorylation may result from reperfusion and reoxygenation in the transplanted kidney. However, this process can also produce large amounts of reactive oxygen species (ROS), causing oxidative stress that damages cells and initiates post-transplantation injury (26). Intriguingly, the mechanisms of oxidative phosphorylation-related genes upregulation are cell type specific. While T cells require mitochondrial ATP from OXPHOS for activation, continued proliferation of the activated T cells relies on OXPHOS (27). This sets precedence for the investigation of T cells and how this may contribute to immunoactivation in TCMR and ABMR.

Anti-HLA DSAs are crucial biomarkers for predicting graft injury and failure. The presence of pre-transplant DSAs or the development of *de novo* DSAs (dnDSAs) is linked to ABMR and graft failure (28). The cellular and molecular pathways regulating ABMR are still under investigation. Evidence suggested that B cell and plasma cell activation leads to the production of DSAs, which can bind to HLA or non-HLA molecules expressed on the endothelial cells of the transplanted kidney (29, 30). Our findings provided a resource atlas for understanding the diverse pathways PT cells utilize in response to DSA+ status, contributing to ABMR after renal transplantation. Although these conditions do not fully recapitulate physiological circumstances in which the DSA contributes to ABMR in PT6 cells, the orientations of these metabolic changes in PT6 cells suggested the rejection status. This indicated that metabolic reprogramming of OXPHOS and other energy utilization influences neutrophil-mediated immunity, myeloid leukocyte-mediated immunity, and granulocyte activation in PT6 during the development of ABMR. Neutrophils rely on diverse metabolic pathways to meet their energy demands and facilitate specialized effector functions, including neutrophil extracellular trap (NET) formation, reactive oxygen species (ROS) production, chemotaxis, and degranulation (31). Moreover, the excessive release of NETs can lead to tissue damage and trigger inflammation (32). Therefore, it is interesting to investigate how this variability in OXPHOS activity impacts the progression of rejection tendency. In addition to the well-known Warburg effect (33), this varied mitochondrial OXPHOS activity also directly supports PT cell activation and neutrophil function.

Our findings indicated that mitochondrial respiration is consistently downregulated across multiple stromal compartments, including PEC and IC-B cells. This notable reduction in oxidative phosphorylation may be linked to the stressed and inflammatory microenvironment within rejection regions, which may suppress mitochondrial electron transport chain (ETC) related genes in stromal cells (34). Interestingly, this metabolic shift is not uniform but varies depending on the ETC complex and cell type. Specifically, our results demonstrated reduced expression of complex I subunits in IC-B cells, decreased expression of complex II subunits, and an broader decline in ETC complex expression within IC-B cells. Moreover, IC-B cells exhibited H+-ATPase localization along the basolateral plasma membrane and in cytoplasmic vesicles. Emerging evidence highlights the innate immune role of ICs, supplementing their traditional function in pH regulation. The oxidative electron flow through the ETC is pivotal for antigen presentation and processing, facilitated by the release of ATP and prostaglandin E2 (PGE2) (35). Notably, our findings revealed for the first time that complex I subunit expression is diminished in IC-B cells during ABMR, suggesting that stromal metabolic dysfunction may contribute to the pathological imbalance observed in ABMR. Our comprehensive analysis of stromal compartments underscores mitochondrial metabolic dysfunction in ABMR, emphasizing that the mechanisms underlying oxidative phosphorylation downregulation are highly cell type specific.

Myeloid cells are a metabolically heterogeneous group of cells that engage in cellular exchange of nutrients and metabolites in the microenvironment. Our work agrees with previous findings and sheds light on metabolic changes and interactions. We found that Myeloid cells increased amino sugar, valine, leucine, isoleucine *in vitro* and that this was regulated by factors secreted from other cells. Thus, we became interested in investigating metabolic exchanges advantageous to MAC.2. For example, previous studies illustrated that macrophages play an injurious role in acute cellular allograft rejection, as well as in chronic injury (36). Understanding the different biological functions of various monocyte/macrophages may help ascertain their role in renal inflammatory response, injury, and repair (37). This phenomenon was demonstrated in our data, driven by IL1-mediated signaling, leading to metabolic rewiring and pro-inflamma- tory response in rejection-associated macrophages.

Cell proportion differences inferred from scRNA-seq data should be interpreted cautiously. The lower proportion of fibroblasts in TCMR and ABMR compared with other conditions may reflect differences in stromal remodeling, sampling region, or the recovery efficiency of matrix-associated cells during tissue dissociation. Similarly, the relative enrichment of PT cells in TCMR and the lower proportion of B cells in ABMR may be influenced by the compositional nature of single-cell data. Another limitation is the relatively small sample size in some groups, particularly the inclusion of only one TCMR biopsy in the discovery cohort. Therefore, TCMR-specific findings should be considered exploratory and hypothesis-generating. Analysis of an independent single-cell/CITE-seq cohort provided supportive evidence for altered T-cell metabolism-related transcriptional programs in TCMR, particularly involving oxidative phosphorylation-related scores. Nevertheless, larger independent single-cell or spatially resolved cohorts will be required to validate PT-specific and other cell-type-specific metabolic remodeling in TCMR. The metabolic programs identified in this study were inferred from transcriptomic data. Direct metabolomic, fluxomic, mitochondrial, and functional assays will be required to determine whether these transcriptional changes correspond to altered biochemical activity.

Our research provided a tissue classification system capturing both cell-type heterogeneity. This paradigm may prove to be an effective pattern for the classification of renal transplantation rejection, may offer fresh perspectives on the metabolism of the condition, identify potential targets for treatment, and highlight important aspects of personalized medicine.

Our research introduced cell-type heterogeneity of renal transplantation rejection, provided novel insights into the metabolic atlas of diverse conditions, and identified potential therapeutic targets.

## Data Availability

The original contributions presented in the study are included in the article/**Supplementary Material**. Further inquiries can be directed to the corresponding authors.

## Supplementary material

Supplementary Figure 1 PT-specific analysis. **(A)** PT UMAP colored by seven-grained cell-state clusters. **(B)**Dot plot displaying critical marker transcripts used to distinguish clusters. **(C)** Proportion of cell clusters for each disease. **(D)** Metabolic pathway activities along with the pseudotime.

Supplementary Figure 2 PT-specific analysis. **(A)**Distribution of fibrosis, inflammation response, and ischemia-reperfusion score on the pseudo-time trajectory. **(B)** Correlation between metabolic pathway activities and fibrosis.

Supplementary Figure 3 Non-PT-specific analysis. **(A)** Non-PT UMAP colored by eleven-grained cell-state clusters. **(B)**Dot plot displaying critical marker transcripts used to distinguish clusters. **(C)** Proportion of cell clusters for each disease.

Supplementary Figure 4 Metabolic alterations in Non-PT cells. **(A)**Metabolic pathways significantly altered in non-PT cells from ABMR samples compared to healthy samples, with corresponding NES and adjusted P-values. **(B)** Schematic representation of valine, leucine, and isoleucine degradation, accompanied by a dot plot showing selected genes involved in their metabolism, comparing healthy kidney samples (black) to ABMR samples (red). **(C)** Schematic of glucose metabolism and the pentose phosphate pathway (PPP), with a dot plot illustrating genes driving glycolysis, showing average expression and percentage expressed in PEC and IC-B cells from ABMR (red) and healthy kidney samples (black). **(D)** Schematic of glutathione metabolism, along with a dot plot visualizing genes involved in glutathione metabolism, displaying average expression and percentage expressed in PEC and IC-B cells from ABMR (red) and healthy kidney samples (black).

Supplementary Figure 5 Myeloid cells-specific analysis. **(A)** Non-PT UMAP colored by seven-grained cell-state clusters. **(B)**Dot plot displaying critical marker transcripts used to distinguish clusters.

Supplementary Figure 6 T cell-specific analysis. **(A)** T cells UMAP colored by seven-grained cell-state clusters. **(B)** Dot plot displaying critical marker transcripts used to distinguish clusters. **(C)** Proportion of cell clusters for each disease. **(D)** Metabolic pathways enriched in TCMR or normal T cells. Significantly enriched pathways with GSEA p-value < 0.05 are highlighted in blue (higher in TCMR) or green (higher in normal samples). **(E)** Dot plot showing the top 16 metabolic pathways.

Supplementary Figure 7 Identification of T cells and comparison of oxidative phosphorylation activity in the GSE189536 dataset. **(A)** Uniform manifold approximation and projection (UMAP) visualization of all cells in the GSE189536 dataset, colored by unsupervised cluster identity. **(B)** UMAP visualization highlighting the annotated T-cell population and all remaining cells. **(C)** Dot plot showing the expression of representative lineage-marker genes across clusters. Dot size indicates the percentage of cells expressing each gene, and color intensity represents the average expression level. **(D)** Violin plots comparing oxidative phosphorylation pathway scores in T cells from non-rejection and T cell–mediated rejection (TCMR) samples. Box plots indicated the median and interquartile range, and individual points represent sample-level values.

Supplementary Figure 8 Evaluation of dataset integration before and after Harmony correction. (A) UMAP visualizations of cells from the four public scRNA-seq datasets before and after Harmony correction, colored by dataset of origin. (B) Violin plots showing the distribution of integration Local Inverse Simpson’s Index (iLISI) values before and after Harmony correction. Box plots indicate the median and interquartile range, and white diamonds indicate the mean. The median iLISI increased from 1.004 to 1.253, and the mean increased from 1.164 to 1.521 after Harmony correction, indicating improved local dataset mixing.

Supplementary Table 1 Information on patient samples.

Supplementary Table 2 Markers of clusters.

Supplementary Table 3 Differentially expressed genes (DEGs) of PT6 cluster (FC > 0.5).
