# Supplementary material for: Single-cell transcriptomic landscape of metabolic reprogramming in kidney allograft rejection
Source: Front Immunol. 2026 Aug 3;17:1755316. doi: 10.3389/fimmu.2026.1755316 (PMC13478174; doi:10.3389/fimmu.2026.1755316)
Supplement: Supplementary file 1 [file Table1.docx]

Table S1: Information on patient samples

| Dataset | Samples | Numbers |
| --- | --- | --- |
| E-MTAB-12051 | Non rejection DSA- | 4 |
|  | Non rejection DSA+ | 8 |
|  | Antibody-mediated rejection | 3 |
|  | T cell-mediated rejection | 1 |
| GSE145927 | Non rejection AKI | 2 |
|  | Antibody-mediated rejection | 3 |
| GSE131685 | Normal | 3 |
| GSE202109 | Normal | 9 |

| **E-MTAB-12051** | Sex | Clinical history | Disease | Time point |
| --- | --- | --- | --- | --- |
| Sample 1 | male | kidney transplant | Non rejection DSA+ | 37 days after transplantation |
| Sample 2 | female | kidney transplant | Antibody-mediated rejection | 61 days after transplantation |
| Sample 3 | male | kidney transplant | Non rejection DSA+ | 12 days after transplantation |
| Sample 4 | female | kidney transplant | Non rejection DSA+ | 91 days after transplantation |
| Sample 5 | male | kidney transplant | Non rejection DSA+ | 9 days after transplantation |
| Sample 5 | male | kidney transplant | Non rejection DSA+ | 9 days after transplantation |
| Sample 7 | male | kidney transplant | Antibody-mediated rejection | 6 days after transplantation |
| Sample 8 | female | kidney transplant | T cell-mediated rejection | 6 days after transplantation |
| Sample 9 | male | kidney transplant | Non rejection DSA- | 91 days after transplantation |
| Sample 10 | male | kidney transplant | Non rejection DSA- | 44 days after transplantation |
| Sample 11 | female | kidney transplant | Non rejection DSA+ | 247 days after transplantation |
| Sample 12 | male | kidney transplant | Non rejection DSA+ | 2104 days after transplantation |
| Sample 13 | male | kidney transplant | Non rejection DSA+ | 118 days after transplantation |
| Sample 14 | male | kidney transplant | Non rejection DSA- | 6 days after transplantation |
| Sample 15 | female | kidney transplant | Non rejection DSA+ | 361 days after transplantation |
| Sample 16 | female | kidney transplant | Antibody-mediated rejection | 2002 days after transplantation |
| **GSE145927** |  |  |  |  |
| Biopsy1 |  |  | Non rejection | 5 days after transplantation |
| Biopsy2 |  |  | Antibody-mediated rejection | 11 days after transplantation |
| Biopsy3 |  |  | Non rejection | 28 days after transplantation |
| Biopsy4 |  |  | Antibody-mediated rejection | 232 days after transplantation |
| Biopsy5 |  |  | Antibody-mediated rejection | 2542 days after transplantation |
